# Vitamin D Deficiency and Glycemic Status in Children and Adolescents with Type 1 Diabetes Mellitus

**DOI:** 10.1371/journal.pone.0162554

**Published:** 2016-09-08

**Authors:** Silvia Savastio, Francesco Cadario, Giulia Genoni, Giorgio Bellomo, Marco Bagnati, Gioel Secco, Raffaella Picchi, Enza Giglione, Gianni Bona

**Affiliations:** 1 Division of Pediatrics, Department of Health Sciences, University of Piemonte Orientale, Novara, Italy; 2 IRCAD (Interdisciplinary Research Center of Autoimmune Diseases), Novara, Italy; 3 Central Laboratory of Maggiore della Carità Hospital, University of Piemonte Orientale, Novara, Italy; 4 Division of Cardiology, Ospedale Santi Antonio e Biagio e Cesare Arrigo, Alessandria, Italy; University of Queensland, AUSTRALIA

## Abstract

**Background:**

Vitamin D (25OHD) effects on glycemic control are unclear in children and adolescents with type 1 diabetes. Aims of this study were to investigate 25OHD status among children with T1DM and its relationship with insulin sensitivity and glycemic status.

**Subjects and Methods:**

A cross sectional study was carried out between 2008–2014. A total of 141 patients had a T1DM >12 months diagnosis and were enrolled in the present study. Of these 35 (24.8%) were migrants and 106 (75.2%) Italians (T2). We retrospectively analyzed data at the onset of the disease (T0)(64 subjects) and 12–24 months before the last visit (T1,124 subjects). Fasting glucose, glycated hemoglobin (HbA1c), 25OHD levels and daily insulin requirement were evaluated and Cholecalciferol 1000 IU/day supplementation for the management of vitamin D insufficiency (<75 nmol/L) was systematically added.

**Results:**

A generalized 25OHD insufficiency was found at each study time, particularly in migrants. At T0, the 25OHD levels were inversely related to diabetic keto-acidosis (DKA) severity (p<0.05). At T1 and T2, subjects with 25OHD ≤25nmol/L (10 ng/mL) showed higher daily insulin requirement (p<0.05) and HbA1c values (p<0.01) than others vitamin D status. The 25OHD levels were negatively related with HbA1c (p<0.001) and daily insulin dose (p<0.05) during follow up. There was a significant difference in 25OHD (p<0.01) between subjects with different metabolic control (HbA1c <7.5%,7.5–8%,>8%), both at T1 and T2. In supplemented subjects, we found a significant increase in 25OHD levels (p<0.0001) and decrease of HbA1c (p<0.001) between T1 and T2, but this was not significant in the migrants subgroup. Multivariate regression analysis showed a link between HbA1c and 25OHD levels (p<0.001).

**Conclusions:**

Children with T1DM show a generalized 25OHD deficiency that impact on metabolic status and glycemic homeostasis. Vitamin D supplementation improves glycemic control and should be considered as an additional therapy.

## Introduction

In recent years, the extra-skeletal effects of vitamin D (25OHD) have raised considerable interest since specific receptors has been found in many tissues and systems, including pancreatic β cells and immune cells. The main extra-skeletal action of vitamin D is exerted into the cell nucleus where it is able to regulate the transcription of approximately the 3% of the human genome [1–3].

25OHD deficiency represents a major health problem since it has been related to cardiovascular, inflammatory, autoimmune diseases, and cancer [4–7]. A high prevalence of 25OHD deficiency has been reported worldwide and especially among migrant populations. Several studies showed that migrants are at higher risk for hypovitaminosis D, suggesting that genetic factors, ethnic origin, and environmental variables such as indoor lifestyle, head covering, poor dietary intake, and lack of supplementation contribute to variability in 25OHD levels [8–10].

Emerging evidences suggest that 25OHD insufficiency may be a risk factor for both type 1 (T1DM) and type 2 diabetes (T2DM). A recent meta-analysis of case-control studies in pediatric patients with T1DM showed 25OHD levels lower than 5.69 ng/ml in patients compared with healthy controls [11]. This reduction seems to be higher in subjects with diabetic ketoacidosis [12–13] and autoantibodies positivity [14]. Moreover, 25OHD status influences glucose homeostasis [15–21]. A study in healthy, non-diabetic adults with normal glucose tolerance showed a positive correlation of 25OHD levels and insulin sensitivity [19]. Furthermore, low 25OHD levels have been associated with insulin resistance in pediatric patients at risk for diabetes and vitamin D and calcium supplementation improved glycemic control in a 12-week study involving children, adolescents and young adults with T1DM [20–21]. Low 25OHD levels seem to produce an inflammatory status in pancreatic islets, leading to a disequilibrium in insulin sensibility and secretion, with consequently insulin resistance and T2DM [15]. Moreover, vitamin D seems to affect glucose homeostasis via a direct effect on β cells and an indirect effect through calcium regulation, since insulin secretion is calcium-dependent [16–17].

However, the impact of 25OHD status on metabolic control and complications in subjects with T1DM is debated and the role of vitamin D supplementation is still unclear [21–22].

Therefore, aims of this study were to investigate 25OHD status among children with T1DM, the relationship between its levels and metabolic homeostasis and the impact of its supplementation on glycemic control.

## Materials and Methods

A cross-sectional study was performed at the Division of Pediatrics, University of Piemonte Orientale (Novara, Piedmont, Italy) between 2008 and 2014. During the study period, a total of 141 patients (mean age 13.3±4.3 years; mean disease duration 5.6±3.9 years; M/F 77/64) had a diagnosis of T1DM > 12 months and were enrolled in the present study. Of these 35 were migrants (25%) and 106 Italians (75%) (T2).

Children with either parents of foreign origin were considered migrants. The 82% of the subjects (116/141) were under intensive insulin treatment in Multiple Dose Insulin injection therapy and the 18% of the patients (25/141) in Continuous Subcutaneous Insulin Infusion.

The diagnosis of T1DM was performed according to the American Diabetes Association criteria [23]. Micro or macro-vascular complications were not present at the enrolment. Ten patients with acute illnesses or under medication interfering with vitamin D metabolism were excluded at enrollment.

Overall, 15% (22/141) of the subjects showed celiac disease and 7% (11/141) had thyroiditis under levothyroxine therapy.

The study was approved from the local Ethical Committee of the “AOU Maggiore della Carità”, Novara, (protocol number 138/14) and informed written consent and assent form was obtained from the patient and parents or next of kin, caretakers, or guardians.

We retrospectively analyzed data at the onset of the disease (T0) (64 subjects) and 12–24 months before the last visit (T1, 124 subjects).

The auxological data and insulin requirement (IU/Kg/day) were collected thought medical records. Breastfeeding duration, formula milk and gluten introductions, vitamin D supplementation in the first year of life, skin photo-types and the ethnic origin were recorded [24].

Fasting glucose, glycated hemoglobin (HbA1c), 25OHD levels, TSH, and free-thyroxine (fT4) were measured at all study times. At the onset, markers of the severity of ketoacidosis (DKA; pH, HCO3-), fasting insulin, c-peptide, diabetes-related autoantibodies (glutamic acid decarboxylase, insulin and protein tyrosine phosphatase islet antigen 2 antibodies), and specific HLA DR/DQ alleles (DR3, DR4, DQ2, DQ8) were evaluated. Children with DRB1*03-DQB1*0201 (DR3/DQ2) or DRB1*04-DQB1*0302 (DR4/DQ8) were categorized as “at high risk” (HLA+).

At T0, the ketoacidosis was defined as severe if pH <7.1 or HCO3 <5 mmol/L, or middle.

At each study time, the subjects were divided according to their 25OHD levels graded as insufficient <75 nmol/L, deficient <50 nmol/L, severely deficient <25 nmol/L according to criteria of Endocrine Society [25].

In addition, at T1 and T2, a sub-analysis was performed dividing all subjects into three groups according to HbA1c levels: <7.5% (better metabolic control), 7.5–8% (tolerable metabolic control), >8% (worse metabolic control).

### Supplementation

The 70% of the subjects had received vitamin D supplementation during the first year of life with 400 IU daily. Cholecalciferol 1000 IU/day supplementation for the management of 25OHD deficiency/insufficiency was systematically added (T0, T1 and T2), as suggested by the Endocrine Society [25]. Subjects adequately supplemented were the 20% at T1 (25/124) and the 67% at T2 (95/141), respectively.

The average duration of supplementation was 17.0±9.5 months at T1 and 17.7±8.1 months at T2.

### Antropometry and assays

The pubertal stages were determined by inspection by a trained physician according to the criteria of Marshall and Tanner. Patients were divided into pre-pubertal and pubertal subjects.

Height was measured by the Harpenden stadiometer and weight by using an electronic scale. Body mass index (BMI) was calculated as body weight divided by squared height (kg/m^2^). Height, weight and BMI-z score were stratified according to growth charts [26].

Plasma glucose levels (mg/dl; 1 mg/dl:0.05551 mmol/liter) were measured by the gluco-oxidase colorimetric method (GLUCOFIX, by Menarini Diagnostici, Florence, Italy). Insulin (μUI/ml; 1 μUI/ml:7.175 pmol/l) was measured by chemiluminescent enzyme-labelled immunometric assay (Diagnostic Products Corporation, Los Angeles, CA). Sensitivity: 2 μUI/ml. Intra- and inter-assay CV ranges: 2.5–8.3 and 4.4–8.6%.

HbA1c levels were measured by the high-performance liquid chromatography (HPLC), using a Variant machine (Biorad, Hercules, CA); intra- and inter-assay coefficients of variation are respectively lower than 0.6 and 1.6%. Linearity is excellent from 3.2% (11 mmol/mol) to 18.3% (177 mmol/mol).

25OHD serum levels (nmol/L) were measured with a direct competitive chemiluminescent immunoassay (Liaison Test 25OHD total, DiaSorin Inc, Stillwater MNUSA). CV for inter- and intra-assay analyses was 10%.

Genomic DNA was extracted from whole blood samples using the kit CeLia-Type (Nuclear Laser Medicine s.r.l). HLA DQ2 and DQ8 typing was performed using the polymerase chain reaction with sequence specific primers (PCR-SSP).

### Statistical Analyses

Data were expressed as mean ± standard deviation (SD), or percentages, as appropriate. For continuous variables, differences were compared using nonparametric Mann-Whitney U test and χ2 test for categorical variables. The evaluation of variation between T1 and T2 for all metabolic parameters was performed with test T for repeated measures. Correlation of 25OHD with continuous values of clinical and biochemical parameters were examined using Pearson correlation coefficients. A partial correlation was used to correct for covariates. Trend evaluation across 25OHD levels was performed though a multinomial regression analyses. Multiple regression analysis has been used to estimate the strength of association among vitamin D and metabolic parameters.

Statistical significance was defined by P values <0.05. All statistical analyses were performed using SPSS for Windows version 17.0 (SPSS, Inc., Chicago, IL, USA).

## Results

All 141 subjects were studied during the last visit (T2) for auxological and metabolic parameters. Data at the onset (T0) and at 12–24 months before the last visit (T1) were analyzed in 64/141 (45%) and 124/141 (87%) patients, respectively. Subjects were both prepubertal and pubertal. The mean age was 7.7±3.9 years at T0, 11.9±4.2 years at T1, and 13.3±4.3 years at T2 (Table 1).

**Table 1:** Characteristics of study population, metabolic parameters and differences in Italian versus migrants at the onset (T0), 12–24 months before the last visit (T1) and at last visit (T2). Data are expressed as mean±SD.

	All	Italian	Migrants	p
	**T0**			
**Number (n,%)**	64 (100)	44 (68)	20 (32)	
**Age (ys)**	7.7±3.9	7.9±4.0	7.0±3.7	**ns**
**Weight (Kg)**	26.3±12.7	27.5±11.5	25.4±10.5	**0.05**
**BMI-zscore**	-0.08±0.911	0.03±0.458	-0.423±0.765	**0.05**
**25OHD (nmol/L)**	44.2±24.0	53.7±22.0	23.2±12.0	**0.0001**
**Insulin (IU/Kg/day)**	0.79±0.29	0.76±0.28	0.90±0.28	**0.05**
**C-peptide (ng/mL)**	0.48±0.46	0.53±0.50	0.34±0.25	**0.05**
**HbA1c (%)**	11.4±2.1	11.4±2.2	11.6±2.1	**ns**
**pH**	7.28±0.15	7.28±0.15	7.27±0.15	**0.05**
**HCO3- (mEq/L)**	17.0±7.4	17.3±7.5	16.1±7.4	**0.05**
	**T1**			
**Number (n, %)**	124 (100)	90 (72)	34 (28)	
**Age (ys)**	11.9±4.2	12.3±4.1	10.6±4.2	**0.05**
**Weight (Kg)**	43.4±17.7	45.7±17.6	36.8±16.4	**0.05**
**BMI-z score**	-0.149±1.191	0.026±0.983	-0.658±1.565	**0.05**
**25OHD (nmol/L)**	46.2±23.0	49.8±22.5	36.4±21.5	**0.0001**
**Insulin (IU/Kg/day)**	0.79±0.26	0.79±0.25	0.81±0.29	**0.05**
**HbA1c (%)**	8.8±1.9	8.5±1.5	9.8±2.6	**0.01**
	**T2**			
**Number (n, %)**	141 (100)	106 (75)	35 (25)	
**Age (ys)**	13.3±4.3	13.7±4.2	12.1±4.3	**0.05**
**Weight (Kg)**	48.5±17.5	50.4±17.2	42.9±17.7	**0.05**
**BMI-z score**	-0.060±0.979	-0.009±0.963	-0.211±1.023	**0.05**
**25OHD (nmol/L)**	56.6±23.7	60.4±23.0	45.2±23.5	**0.0001**
**Insulin (IU/Kg/day)**	0.78±0.24	0.78±0.23	0.79±0.26	**0.05**
**HbA1c (%)**	8.3±1.3	8.1±1.2	9.1±1.5	**0.01**

Auxological and metabolic parameters at the three study times are shown in Table 1.

In the first year of life, Italian patients showed an earlier introduction of formula milk than migrants (2.3±1.6 vs 3.2±1.9 months; p<0.05) and although not significant shorter breastfeeding duration, (6.9±4.0 vs 9.5±5.0 months).

At T0, migrants presented lower c-peptide (p<0.05) and 25OHD levels (p<0.0001) than Italian with a higher insulin requirement at discharge (p<0.05). Migrants had an earlier age at the onset, although not significant. No differences were found in HbA1c levels (Table 1).

At both T1 and T2, migrants had a lower age, weight, BMI-z score (p<0.05), and 25OHD levels (p<0.0001) compared to Italian and a higher HbA1c (p<0.01) and insulin requirement (p<0.05) (Table 1).

We found 25OHD insufficiency in 26.6% of subjects, deficiency in 40.6% and severely deficiency in 23.4%. Only 9.4% of the cases showed sufficient 25OHD levels and all these subjects were Italian (Table 2).

**Table 2:** 25OHD levels (nmol/L) in the whole population and differences in Italian versus migrants at the onset (T0), 12–24 months before the last visit (T1) and at last visit (T2). Data are expressed as number of subjects and percentage (%).

25OHD levels (nmol/L)	All	Italian	Migrants	p^for trend^
**T0**				
**Number of subjects (n, %)**	64 (100)	44 (68)	20 (32)	
**x≥75 n (%)**	6 (9.4)	6 (13.6)	0	
**50≤x<75 n (%)**	17 (26.6)	17 (38.6)	0	<0.0001
**25≤x<50 n (%)**	26 (40.6)	19 (43.2)	7 (35.0)	
**<25 n (%)**	15 (23.4)	2 (4.5)	13 (65.0)	
**T1**				
**Number of subjects (n, %)**	124 (100)	90 (72)	34 (28)	
**x≥75 n (%)**	13 (10.5)	11 (12.2)	2 (5.9)	
**50≤x<75 n (%)**	38 (30.6)	33 (36.7)	5 (14.7)	<0.05
**25≤x<50 n (%)**	45 (36.3)	30 (33.3)	15 (44.1)	
**<25 n (%)**	28 (22.6)	16 (17.8)	12 (5.3)	
**T2**				
**Number of subjects (n, %)**	141 (100)	106 (75)	35 (25)	
**x≥75 n (%)**	29 (20.6)	25 (23.6)	4 (11.4)	
**50≤x<75 n (%)**	56 (39.7)	44 (41.5)	12 (34.3)	<0.001
**25≤x<50 n (%)**	44 (31.2)	34 (32.1)	10 (28.6)	
**<25 n (%)**	12 (8.5)	3 (2.8)	9 (25.7)	

At T0, subjects who showed 25OHD levels <25 nmol/L presented a more severe DKA compared to patients with 25OHD >25 nmol/L (pH 7.23±0.16 vs 7.31±0.13; HCO3- 14.2±7.8 vs 18.1±7.0 mEq/L; p<0.05, respectively) and a greater insulin requirement at discharge (0.98±0.24 vs 0.77±0.24 IU/kg/day; p<0.01).

Variability in pH and bicarbonates values (p<0.01) across 25OHD levels was observed (Table 3).

**Table 3:** Metabolic parameters and insulin requirement at the onset (T0), 12–24 months before the last visit (T1) and at last visit (T2) across 25OHD levels according to Endocrine criteria. Data are expressed as mean±SD. 25OHD sufficiency (S), insufficiency (I), deficiency (D), severe deficiency (SD).

	25OHD >75 nmol/L (S)	25OHD 50–75 nmol/L (I)	25OHD <50 nmol/L (D)	p ^for trend^	25OHD <25 nmol/L (SD)
**T0**					
**pH**	7.28±0.85	7.37±0.16	7.26±0.13	**0.01**	7.23±0.16
**Bicarbonates (mEq/L)**	14.2±6.9	21.1±5.6	16.1±7.5	**0.05**	14.1±7.7
**c-peptide (ng/mL)**	0.25±0.15	0.45±0.51	0.31±0.2	**ns**	0.22±0.22
**25OHD (nmol/L)**	93.6±13.7	62.1±7.5	29.5±6.5	**<0.0001**	16.8±5.7
**Insulin (IU/Kg/day)**	0.91±0.28	0.73±0.24	0.85±0.2	**ns**	0.98±0.24
**HbA1c (%)**	12.0±1.0	11.3±2.1	11.4±1.9	**ns**	12.2±1.8
**T1**					
**25OHD (nmol/L)**	89±14	61.5±6.5	30.25±11.7	**<0.0001**	18.1±3.75
**Insulin (IU/Kg/day)**	0.76±0.27^e^	0.73±0.25^e^	0.83±0.26^e^	**ns**	0.95±0.29^e^
**HbA1c (%)**	7.4±0.6^a^^,^^f^	8.3±1.7^b^^,^^f^	9.2±2.0^a^^,^^b^^,^^f^	**<0.0001**	9.8±2.2^f^
**T2**					
**25OHD (nmol/L)**	90.2±14.7	62±7.0	33.7±10.2	**<0.0001**	19.2±3.7
**Insulin (IU/Kg/day)**	0.71±0.25^c^^,^^e^	0.76±0.20^e^	0.83±0.21^c^^,^^e^	**0.05**	0.91±0.22^e^
**HbA1c (%)**	8.0±1.2^d^^,^^f^	8.1±1.25^d^^,^^f^	8.7±1.3^d^^,^^f^	**0.01**	9.8±1.6^f^

^a^ = p<0.0001 D vs S at T1

^b^ = p<0.01 D vs I at T1

^c^ = p<0.05 D vs S at T2

^d^ = p<0.01 D vs S and D vs I at T2

^e^ = p<0.05 SD vs other 25OHD status at T1 and T2

^f^ = p<0.01 SD vs other 25OHD status at T1 and T2

At T1 and T2, the 10.5% and the 20.6% of the subjects showed 25OHD sufficiency, the 30.6% and the 39.7% had an insufficiency, the 36.3% and the 31.2% showed a deficiency, and the 22.6% and 8.5% had a severely deficiency (Table 2).

At both T1 and T2, migrants had a lower age, weight, BMI-z score (p<0.05), and 25OHD levels (p<0.0001) compared to Italian and a higher HbA1c (p<0.01) and insulin requirement (p<0.05) (Table 1).

At T1, children with deficient 25OHD status had HbA1c values higher than sufficient (p<0.0001) and insufficient (p<0.01) subjects; at T2, patients with 25OHD levels <50 nmol/l showed a higher daily insulin requirement (p<0.05) than sufficient and HbA1c levels higher (p<0.01) than sufficient and insufficient subjects. Not significant differences between sufficient and insufficient subjects were found both at T1 and T2; moreover, in patients with a severe 25OHD deficiency, a higher daily insulin requirement (p<0.05) and HbA1c values (p<0.01) than other 25OHD status were found and a trend to an increase in HbA1c (p<0.0001) across 25OHD levels was observed at T1 and in HbA1c (p<0.01) and insulin requirement (p<0.05) at T2 (Table 3).

**Fig 1:**
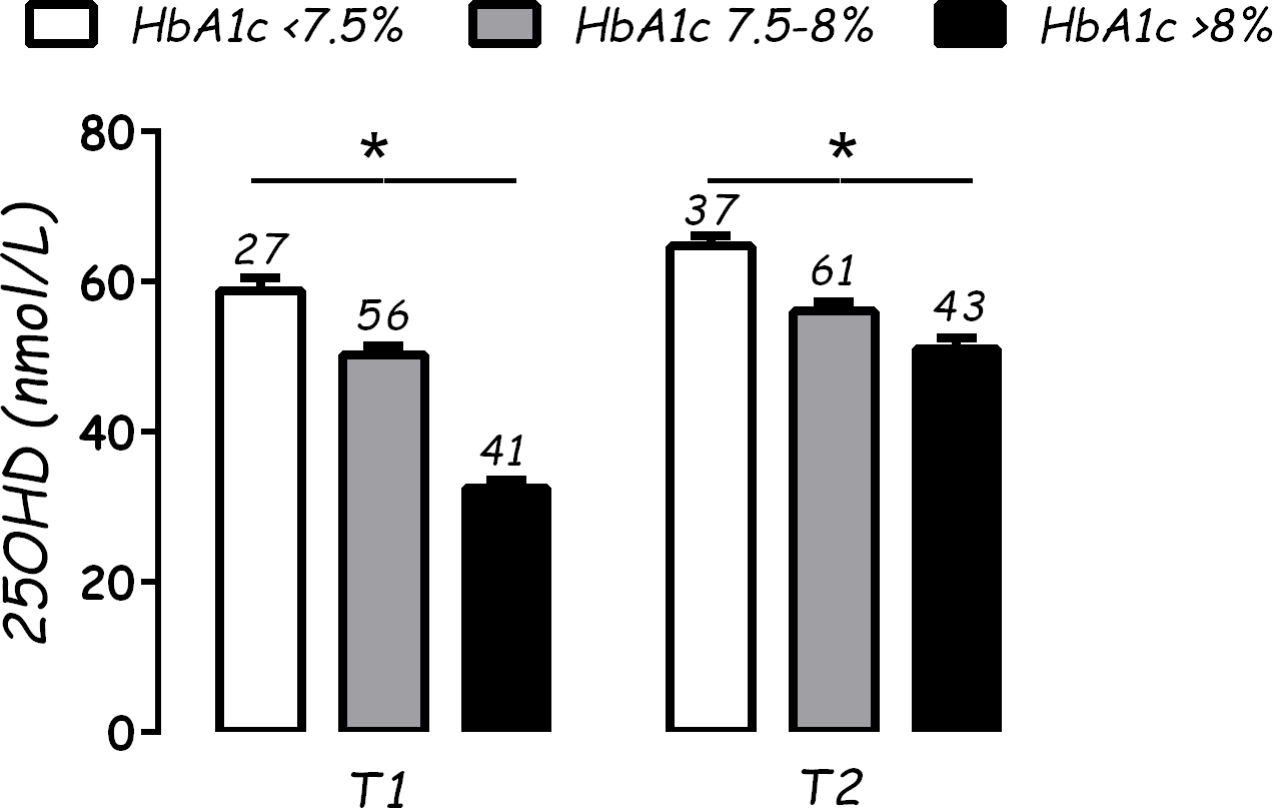
Vitamin D (25OHD) levels (nmol/L) according to glycated hemoglobin (HbA1c) levels (%). On each bar graph are reported the number of subjects (n); * = p<0.01.

Analyzing the subjects according to HbA1c status (<7.5%, 7.5–8%, >8%), there was a significant difference in 25OHD (p<0.01) between subjects with better metabolic control and the others, both at T1 and T2 (Fig 1). No differences for sex or HLA status were found.

### The impact of vitamin D supplementation

All patients with serum 25OHD less than 75 nmol/l were supplemented with cholecalciferol 1000 IU/day. Children under supplementation from T0, at T1 showed a significantly higher 25OHD level (57.8±23.3 vs 43.2±22.0 nmol/L; p<0.01), lower HbA1c (8.1±1.1 vs 9.0±2.0%; p<0.05), and lower daily insulin requirement (0.68±0.22 vs 0.81±0.26 IU/Kg/day; p<0.05) than not supplemented.

Significant changes in 25OHD levels (p<0.0001) and HbA1c (p<0.001) between T1 and T2 were found only in supplemented subjects.

**Fig 2:**
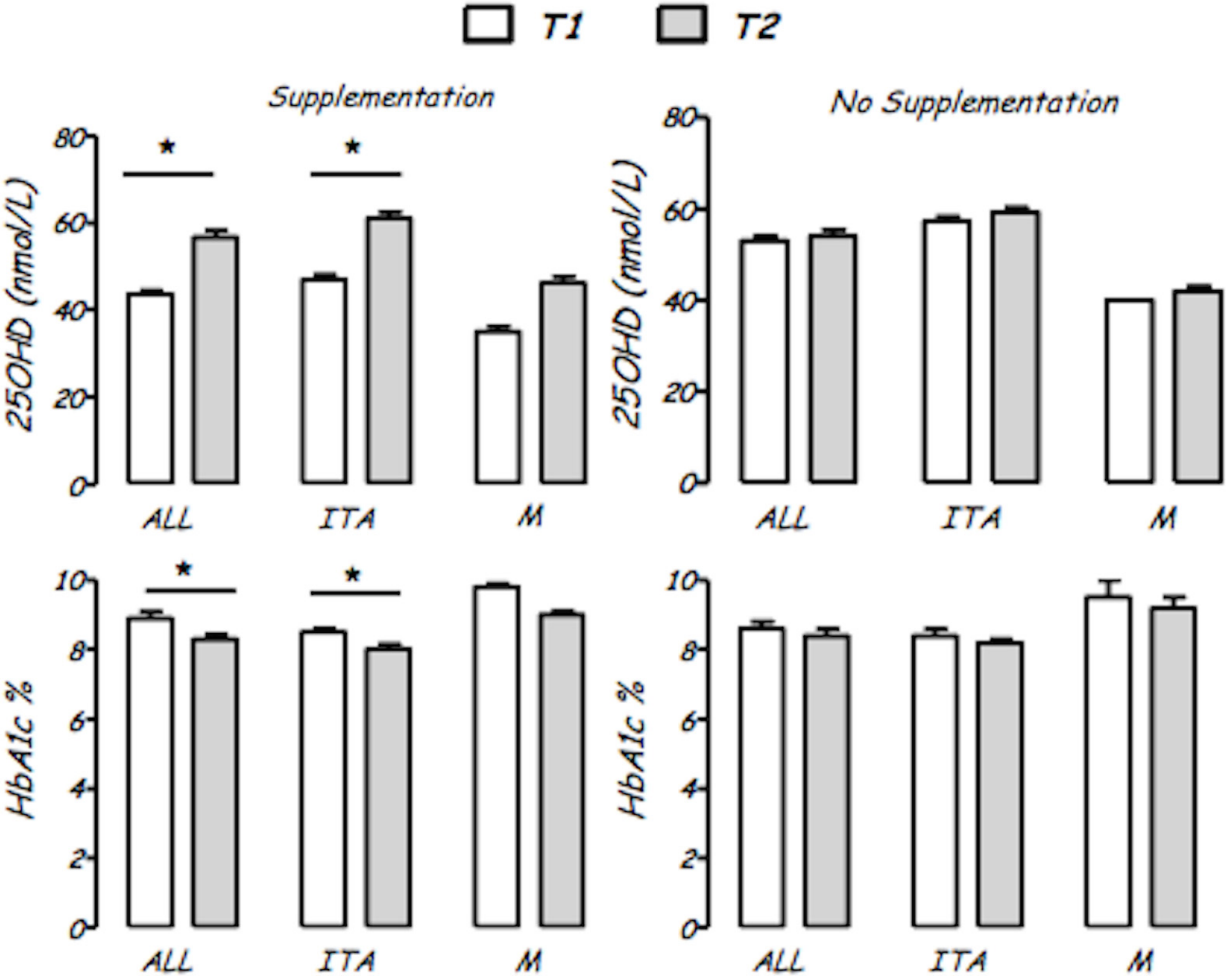
Vitamin D (25OHD, nmol/L) and glycated hemoglobin (HbA1c, %) levels in the whole population (ALL), in Italian (ITA) and in migrant (M) subjects according to vitamin D supplementation. * = p < 0.001 compared to T1.

Analyzing data by ethnicity and presence/absence of supplementation, the same statistical evidences were present only in Italian subjects (Fig 2).

### Correlations

At T0, 25OHD levels were related with pH (r:0.326, p<0.01) and bicarbonates concentration (r:0.270, p<0.05). HbA1c values showed an association with pH (r:-0.180, p = 0.05), c-peptide (r:-0.290, p = 0.002) and insulin daily requirement (r:0.367, p<0.0001). At T1, 25OHD status was negatively related with HbA1c (r:-0.389, p<0.0001) and daily insulin dose (r:-0.307, p<0.01), also when corrected for BMI z-score and puberty. HbA1c values were associated with BMI z-score (r:0.408, p<0.0001) and insulin requirement (r:0.227, p<0.01).

**Fig 3:**
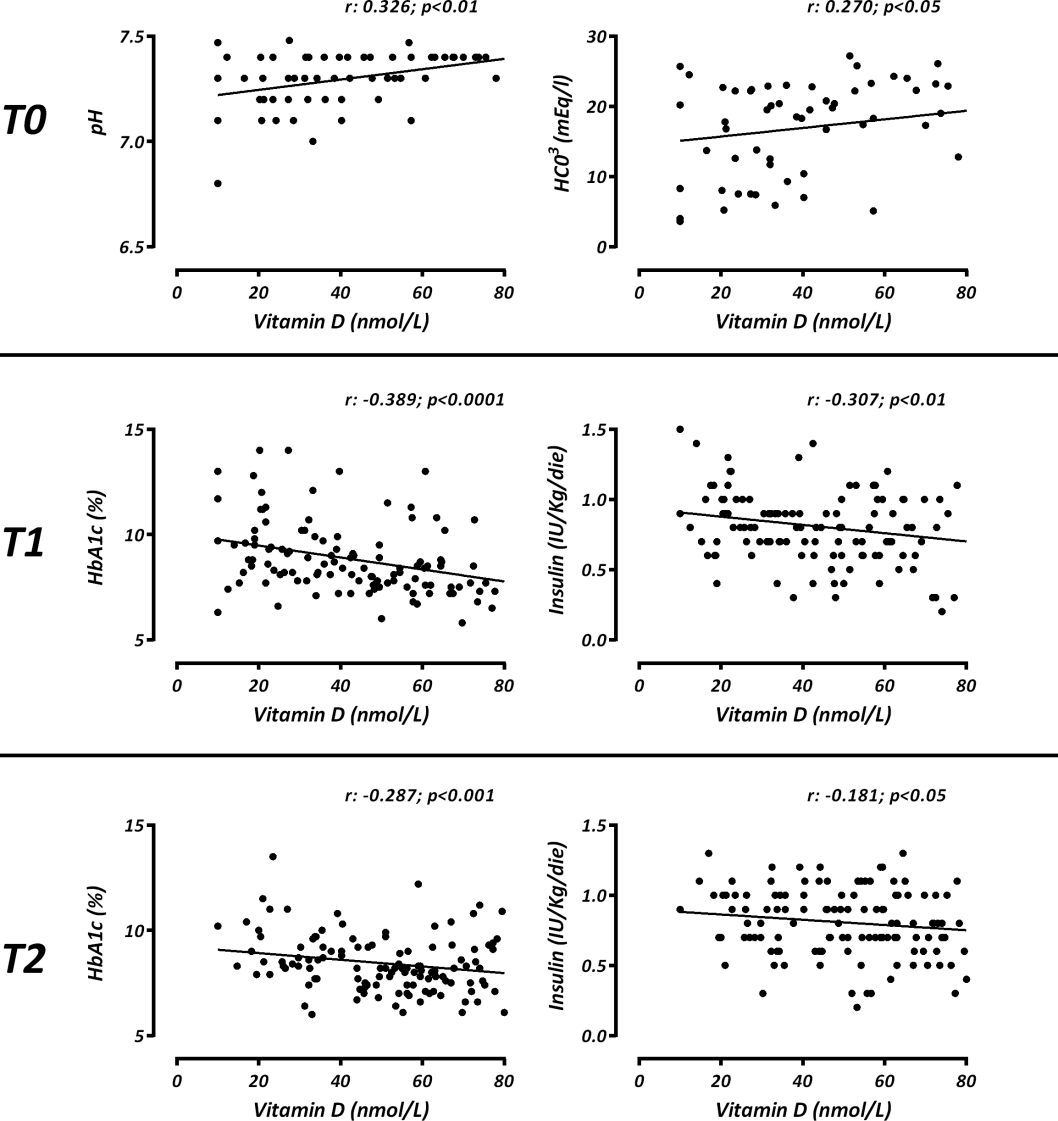
Correlation for Vitamin D (25OHD, nmol/L) with metabolic/ biochemical parameters in all subjects at three times (T0, T1, T2).

At T2, 25OHD values were negatively related with HbA1c (r:-0.287, p<0.001) and daily insulin dose (r:-0.181, p<0.05), also when corrected for BMI z-score, as at T1 (Fig 3).

A multivariable regression model analysis (R-square: 0.078) showed a link between the dependent variable HbA1c and the independent variable 25OHD levels (β:-0.291; p<0.001) adjusted for age, BMI-z score and phototype.

## Discussion

The study aimed to investigate the 25OHD status in children with T1DM, and the relationship with glycemic control at disease’s onset (T0) and in the subsequent stages of routine therapy (T1 and T2). The secondary outcome was to assess a possible therapeutic use of vitamin D to improve clinical and metabolic control in childhood T1DM at onset and at follow up.

In our pediatric series with T1DM living in north Italy (45° north parallel) we found a high prevalence of hypovitaminosis D, thus at the onset of T1DM only the 9.4% showed sufficient 25OHD levels accordingly to the Endocrine criteria (>75 nmol/L) [25].

This finding confirms the widespread hypovitaminosis D in the pediatric population with T1DM, particularly at the onset of the disease [11, 13, 27–28] as recently suggested by a case-control studies “meta-analysis” of pediatric subjects with T1DM [11].

In the present study we found a difference between migrants and Italian children, the first showing a worst 25OHD deficiency, related to a more severe DKA at onset. This observation may account the results of a previous multicenter study in Italy [29] and suggests the role of 25OHD deficiency as environmental factor triggering the development of diabetes and worsening the clinical presentation at onset. Indeed, as previously shown, the reduction of 25OHD levels seems to be higher in subjects with diabetic keto-acidosis at presentation [12–13].

Our data show that a severe onset of T1DM could be prevented by normal 25OHD level, hypothetically through a slower decline of β cells function due to a specific immunomodulatory action of vitamin D, as suggested by a recent study showing that vitamin D3, given as a therapy to patients at the onset of T1DM, had positive effects on cytokine, regulatory T cells levels and residual β-cell function [30]. Moreover, the immunological function of vitamin D has been recently reported in healthy adults treated with 3 months administration of cholecalciferol (140.000 IU/month vs placebo) [31].

During the follow-up, a severe hypovitaminosis D was associated to a worse HbA1c and to higher insulin requirement, but this metabolic effect cannot be explained by an immune role as previously. Vitamin D may act directly on β cell, promoting insulin secretion and regulating calcium homeostasis or through a peripheral action, increasing the insulin sensitivity of target cells, or regulating the activation of several calcium-dependent enzymes involved in glucose metabolism [17, 30, 32].

A study in healthy, non-diabetic adults with normal glucose tolerance showed a positive correlation of 25OHD and insulin sensitivity: the rise in 25OHD from 25–80 nnoml/l might increase insulin sensitivity by 60%, suggesting as vitamin D supplementation could be an adjunctive therapy in diabetes mellitus [33]. Furthermore, epidemiological studies, in patients with T2DM show a significant relationship between low 25OHD levels and reduced glucose tolerance [34–36].

From out of our knowledge, to now, few study investigated 25OHD status and glucose metabolism in childhood with T1DM. Tunc et al., showed that 25OHD levels were significantly lower and insulin requirement higher in patients with T1DM and poor metabolic control [37].

The second aim was to study the effect of vitamin D supplementation on glycemic status. In our study, Italian patients under vitamin D supplementation exhibited a better metabolic control and a lower insulin requirement than not supplemented children, suggesting a role of vitamin D treatment in glycemic control and insulin sensitivity. To now, only few studies analyzed the role of vitamin D treatment on glycemic control in subjects with T2DM and T1DM and their results are still conflicting [22, 36, 37–39]. A recent double-blinded trial showed that cholecalciferol supplementation resulted in a protective immunologic effect and in a slow decline of residual β-cell function [30]. Furthermore, Ordooei et al., found that vitamin D administration leads to a decrease of fast blood sugar and HbA1c levels in children and adolescents without affecting calcium [40] and the IMDIAB study showed a reduction in insulin doses in children and adolescents with newly diagnosed T1DM treated for 1 year with calcitriol (0.25 mcg/2 days) vs those who were treated with nicotinamide (25 mg/kg/day) [41]. In contrast, the elegant study of Nwosu and Maranda showed a significant reduction of HbA1c after 3 months of ergocalciferol or cholecalciferol supplementation in children and adolescents with T2DM but not in subjects with T1DM [36] and the IMDIAB XIII study did not found differences in dose of insulin, HbA1c and C-peptide at 6, 12, 24 months of treatment with calcitriol (0.25 mcg/day) vs placebo in adolescents and young adults with newly diagnosed T1DM and high baseline levels of C-peptide [42].

The impact of BMI on the relationship between 25OHD concentration and insulin sensitivity has been proved by several studies that showed a strong correlation between overweight and hypovitaminosis D suggesting a potential role of supplementations in this subgroup and an improvement of insulin sensitivity and BMI [33, 36, 43–44]. In our study, all subjects were normal weight and the BMI did not correlate with insulin requirement and 25OHD levels at all study stages.

Additionally, the migrant status, can impact on 25OHD levels and requirements. Accordingly, our study showed a trend to a reduction of HbA1c in migrants subgroup treated with vitamin D. From one hand the lack of statistical significance might be explained by our small sample size, while from the other this might be explained by the higher vitamin D dose eventually required in this subset of population.

There is still no consensus about the best dose of supplementation [25]. In this study, although most of patients were consuming vitamin D, at the endpoint only 29/141 had sufficient 25OHD levels, according to the Endocrine criteria. Probably we need to consider ethnic differences among individuals to define what the correct intake of vitamin D. Surely, lifestyle variables, as clothing, outdoor sports activities, the food choice and socio-economic status can impact on 25OHD levels. These variables were not investigated and this represents a limitation of this study. A prospective case-control double-blind study must be planned to see if the vitamin D supplementation really produces a better metabolic control of T1DM in children. Despite the limits of this work, a 25OHD deficiency correction program should be considered.

In conclusion, 25OHD deficiency is widely present in children with T1DM and seems to impact on metabolic status and glycemic homeostasis. The possible role of vitamin D supplementation, as an additional therapy, to increase glycemic control and insulin sensitivity opens new perspectives to increase the control of the disease and to improve health of these patients.
